# Determination of the Cannabinoid CB1 Receptor’s Positive Allosteric Modulator Binding Site through Mutagenesis Studies

**DOI:** 10.3390/ph17020154

**Published:** 2024-01-24

**Authors:** Hayley M. Green, Daniel M. J. Fellner, David B. Finlay, Daniel P. Furkert, Michelle Glass

**Affiliations:** 1 Department of Pharmacology and Toxicology, School of Biomedical Sciences, University of Otago, Dunedin 9016, New Zealand; hayley.green@postgrad.otago.ac.nz (H.M.G.); david.finlay@otago.ac.nz (D.B.F.); 2 School of Chemical Sciences, Faculty of Science, University of Auckland, Auckland 1142, New Zealand; dfel694@aucklanduni.ac.nz (D.M.J.F.); d.furkert@auckland.ac.nz (D.P.F.); 3 Maurice Wilkins Centre for Molecular Biodiscovery, The University of Auckland, Auckland 1142, New Zealand

**Keywords:** Cannabinoid CB1 receptor, allosteric modulation, allosteric agonism, GAT229, GAT228, ZCZ011, binding site

## Abstract

Positive allosteric modulators (PAMs) of the cannabinoid CB1 receptor (CB_1_) offer potential therapeutic advantages in the treatment of neuropathic pain and addiction by avoiding the adverse effects associated with orthosteric CB_1_ activation. Here, molecular modeling and mutagenesis were used to identify residues central to PAM activity at CB_1_. Six putative allosteric binding sites were identified in silico, including novel sites previously associated with cholesterol binding, and key residues within each site were mutated to alanine. The recently determined ZCZ011 binding site was found to be essential for allosteric agonism, as GAT228, GAT229 and ZCZ011 all increased wild-type G protein dissociation in the absence of an orthosteric ligand; activity that was abolished in mutants F191A^3.27^ and I169A^2.56^. PAM activity was demonstrated for ZCZ011 in the presence of the orthosteric ligand CP55940, which was only abolished in I169A^2.56^. In contrast, the PAM activity of GAT229 was reduced for mutants R220A^3.56^, L404A^8.50^, F191A^3.27^ and I169A^2.56^. This indicates that allosteric modulation may represent the net effect of binding at multiple sites, and that allosteric agonism is likely to be mediated via the ZCZ011 site. This study underlines the need for detailed understanding of ligand receptor interactions in the search for pure CB_1_ allosteric modulators.

## 1. Introduction

The cannabinoid CB1 receptor (CB_1_) is the most abundant G protein-coupled receptor (GPCR) in the brain, where CB_1_ acts as a synaptic circuit breaker for hyperexcitability by decreasing neurotransmitter release [1]. Classical activation of CB_1_ occurs when a ligand binds to the endogenous ligand (orthosteric) binding site, causing a conformational change in the receptor to allow guanine nucleotide exchange and dissociation of a heterotrimeric G protein. Canonically, CB_1_ couples to inhibitory G_α_ proteins (G_αi/o_). Following dissociation of the heterotrimeric G protein, the active G_α_ subunit inhibits adenylate cyclase-mediated production of cyclic adenosine monophosphate (cAMP), increases the phosphorylation of extracellular signal-related kinase 1/2 (ERK1/2), and changes the polarisation of the cell by modulating potassium and calcium channels. Targeting CB_1_ has proven to be promising in the treatment of neurodegenerative and pain-related disorders; however, therapeutic utility is limited by on-target adverse effects, such as catalepsy, tolerance, and dependence [2,3,4].

Allosteric modulation of CB_1_ is an alternative approach to targeting the endocannabinoid system. Allosteric modulators are compounds that bind to a site that is topographically distinct from the orthosteric binding site and can increase (positive) or decrease (negative) orthosteric ligand binding affinity and/or signalling efficacy [5]. Allosteric modulators are typically inactive in the absence of orthosteric ligands and are therefore hoped to produce fewer on-target adverse effects, as they modulate endogenous cannabinoid signalling, maintaining the endocannabinoid spatiotemporal signalling pattern, and limit global receptor activation [6]. The first compounds with reported positive allosteric modulator (PAM) activity at CB_1_ were the *N*-alkyl and bi-aryl substituted tropanes RTI-370, RTI-371, JHW-007, and substituted piperazine GBR-12909 [7]. The second series of PAMs characterised at CB_1_ are 2-phenylindole compounds ZCZ011 and GAT211, which are both racemic mixtures of two enantiomers formed due to the chiral carbon at the centre of the compound [8,9]. In addition to showing some PAM activity, both ZCZ011 and GAT211 are also allosteric agonists at CB_1_ because they stabilise active receptor conformation and cause both G protein signalling and β-arrestin translocation to CB_1_ [8,9,10]. Positive allosteric modulators of CB_1_, including GAT211, ZCZ011, and other structural analogues, have been found to decrease neuropathic pain, intraocular pressure, and opioid addiction in the absence of on-target adverse effects [8,11,12,13,14,15,16,17,18], supporting the hypothesis that allosteric activation may show improved therapeutic potential. In light of this, a more thorough understanding of their mechanism of action will further enhance the potential utility of PAMs at CB_1_.

Despite showing promising therapeutic effects in vivo, CB_1_ PAMs show low potency and have both poor solubility and poor metabolic stability [14,19,20]. Consequently, structural activity relationship studies have sought to enhance the drug-like features of CB_1_ PAMs. Using the GAT211 scaffold, the replacement of hydrogen atoms with fluorine and/or nitrogen at certain positions either abolished or augmented the allosteric agonism and/or PAM effects of GAT211 [20]. Trifluorination of GAT211 was found to increase the potency and efficacy of allosteric agonism, which was investigated by using cAMP inhibition experiments in HEK293 cells [20]. Trifluorination was also suggested to increase the PAM effects of GAT211, shown as an increase in efficacy and potency of the orthosteric ligand CP55940, although the data in this manuscript are more consistent with an increase in allosteric agonism with no change in allosteric modulation [20]. The nitro group is often regarded as a toxicophore and is thus avoided in medicinal chemistry [21]. However, relatively little data exist on aliphatic nitro groups. Using ZCZ011 as a scaffold, it has been shown that replacing the NO_2_ with a trifluoromethyl group resulted in increased metabolic stability in both rat and human liver microsomes, while the signalling profile of ZCZ011 was retained [14].

Resolution of racemic GAT211 into its enantiomers ((*R*)-GAT229 and (*S*)-GAT228) led to the suggestion that the *R*-enantiomer (GAT229) is a “pure” allosteric modulator with no intrinsic efficacy, while the *S*-enantiomer (GAT228) is an allosteric agonist, as it causes activation of CB_1_ in the absence of orthosteric ligand [9]. This difference has been attributed to each enantiomer binding to distinct putative binding sites, with GAT229 binding to a site that results in positive allosteric modulation and GAT228 binding to a site that results in receptor activation [20,22]. Complicating this interpretation, we have recently shown that GAT229 is an efficacious allosteric agonist, therefore indicating a binding interaction that results in receptor activation (Green et al., 2023—submitted). In addition, the crystal structure of CB_1_ bound by the orthosteric agonist CP55940 and the PAM (*S*)-ZCZ011 was recently solved, indicating that this ligand binds to a site distinct from either of the putative GAT229 or GAT228 binding sites [23].

Increased understanding of the binding site(s) of PAMs at human CB_1_ (hCB_1)_ will facilitate the design of novel compounds to elicit specific outcomes in CB_1_ potency and/or its efficacy of downstream signalling. This study aimed to probe the putative binding sites of PAMs by mutating key residues within both proposed and novel binding sites within hCB_1_ to identify residues crucial for PAM and allosteric agonist signalling.

## 2. Results

### 2.1. Identification of Putative PAM Binding Sites

**Figure 1:**
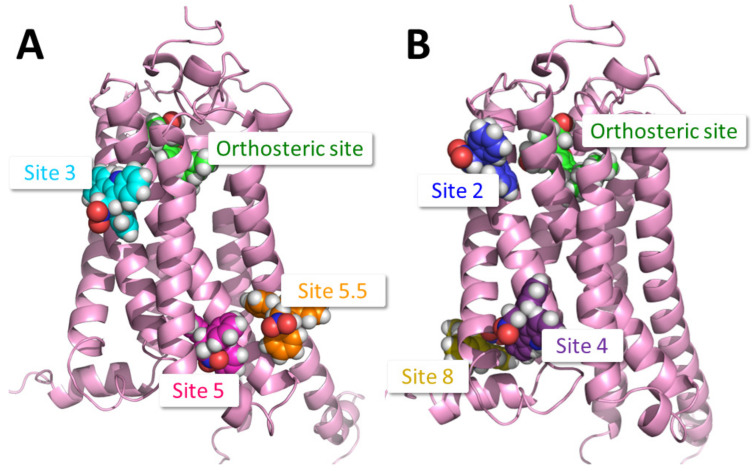
The six putative PAM binding sites on CB_1_ considered in this study, shown with bound GAT229, and the orthosteric agonist CP55940 (green). (**A**) View of Site 3, the ZCZ011 binding site from the crystal structure in [23] (GAT229 in cyan), and two potential cholesterol binding sites, Site 5 (GAT229 in magenta), and Site 5.5 (GAT229 in orange). (**B**) View of Site 2, the putative GAT229 binding site proposed in [22] (GAT229 in blue), Site 4, the putative GAT228 binding site proposed in [22] (GAT229 in purple), and Site 8, the putative pregnenolone binding site proposed in [24] (GAT229 in yellow). [Figure created using the PyMOL Molecular Graphics System, Version 2.0 Schrödinger, LLC New York, NY, USA].

**Figure 2:**
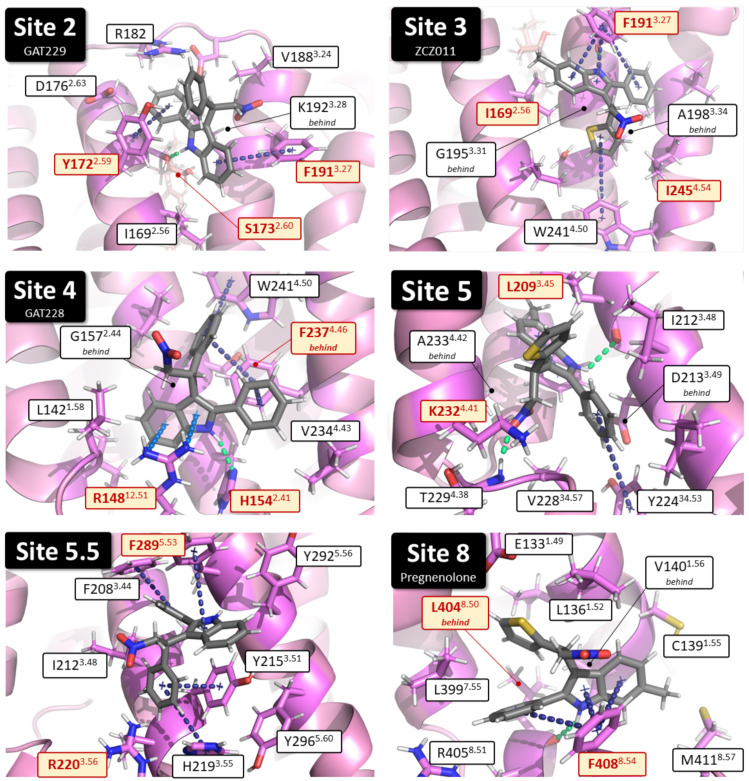
Individual binding details for the six putative PAM CB_1_ binding sites in hCB_1_. Residues mutated in this study are highlighted in yellow (see Supplementary Material for full interaction energy tables of binding site residues). Compounds shown docked to hCB_1_ in each image are **Site 2**, GAT229; **Site 3**, (*S*)-ZCZ011; **Site 4**, GAT228; **Site 5**, (*S*)-ZCZ011; **Site 5.5**, GAT228; **Site 8**, (*R*)-ZCZ011.

Utilising the approach described in Section 4.3, nine potential binding sites were identified. Out of the nine putative binding sites, the six most likely binding sites were chosen to be investigated in the mutagenesis study (Figure 1). Within these six putative binding sites, per-residue interaction energy analysis indicated the residues most important for binding (see Table 1, Figure 2, and Supplementary Material for full data), whereby interaction energies for residues within each putative binding site were reported, with high interaction energies indicating an increased likelihood for involvement in allosteric binding and/or activation (see Table 1, Figure 2, and Supplementary Material for full data). For each putative binding site, the two or three residues with the highest interaction energies were identified as important in allosteric ligand binding (Table 1) and each was mutated to alanine as described below (Figure 2). Residues that were identified as crucial in receptor activation were excluded from mutagenesis studies.

### 2.2. Effect of Mutating Key hCB_1_ Residues on Orthosteric and Allosteric Agonist/Inverse Agonist-Induced G Protein Dissociation

To gain insight into the binding sites of allosteric modulators at CB_1_, key interacting residues within six putative binding sites were identified and mutated to an alanine. If the ligand interacts with one of the mutated residues in WT CB_1_ upon binding, a mutation at this site should result in decreased G protein dissociation. To understand the effect of each receptor mutant on allosteric ligand G protein dissociation, we first evaluated the effect of mutations within putative allosteric binding sites on orthosteric agonist (CP55940)-induced G protein dissociation.

**Figure 3:**
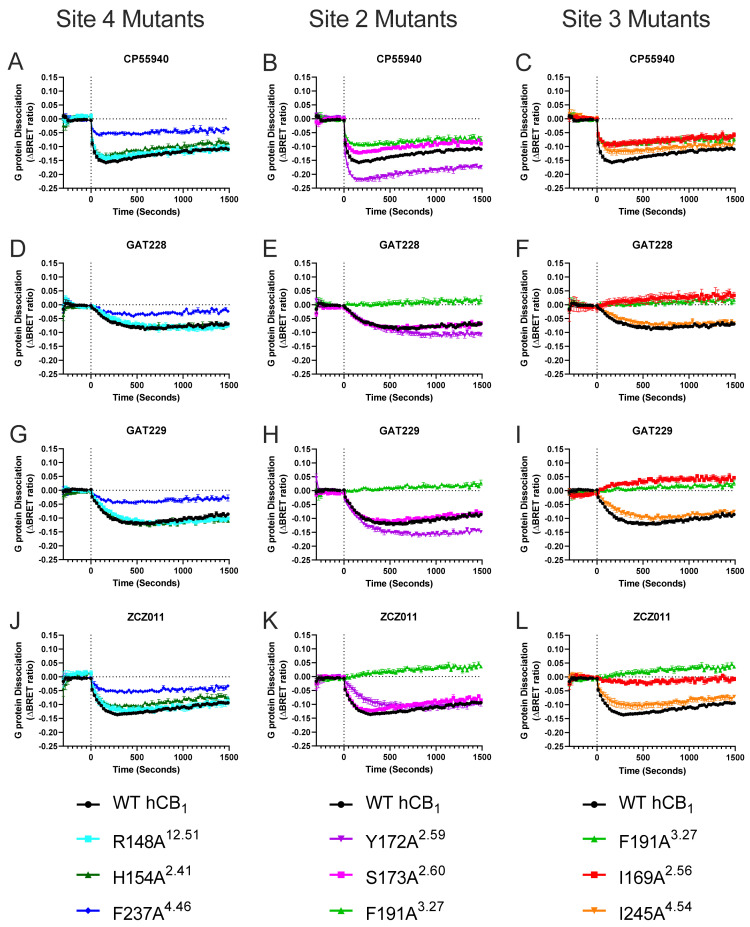
G_αi3_ protein dissociation by 10 μM cannabinoid ligands in HEK293 cells transiently transfected with WT hCB_1_ or mutant receptor. Kinetic traces comparing G_αi3_ protein dissociation by cannabinoid ligands at WT hCB_1_ (black curves) to Site 4 mutants (putative GAT228 binding site; (**A**,**D**,**G**,**J**)), Site 2 mutants (putative GAT229 binding site; (**B**,**E**,**H**,**K**)), and Site 3 mutants (putative ZCZ011 binding site; (**C**,**F**,**I**,**L**)) over a 25 min period. Data are pooled across five independent biological replicates and expressed as mean ± SEM. Data are expressed as ΔBRET ratio as matched vehicle conditions have been subtracted.

**Figure 4:**
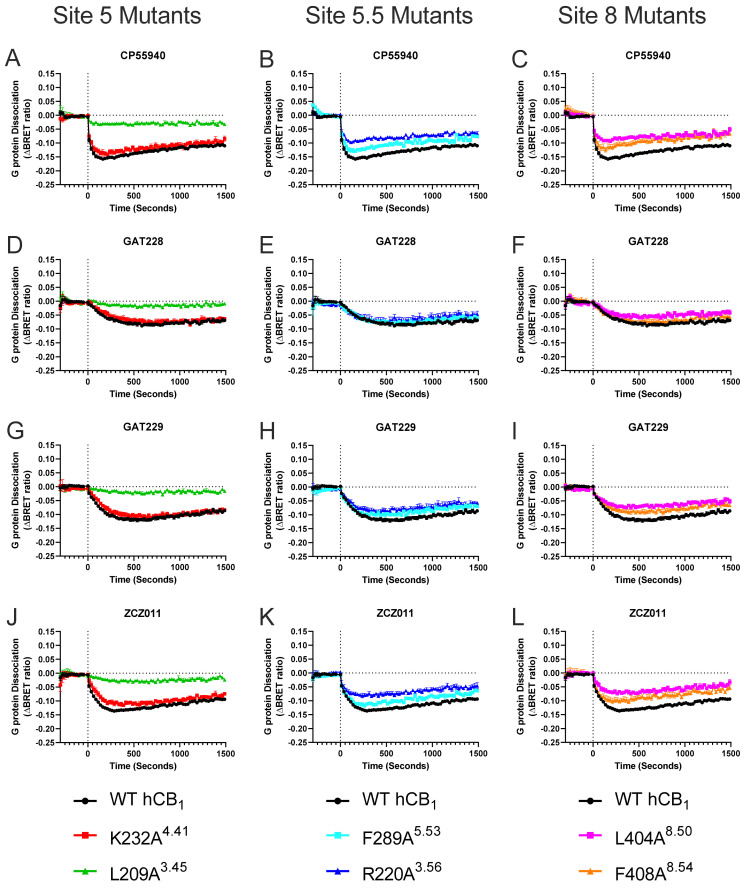
G_αi3_ protein dissociation by 10 μM cannabinoid ligands in HEK293 cells transiently transfected with WT hCB_1_ or mutant receptor. Kinetic traces comparing G_αi3_ protein dissociation by cannabinoid ligands at WT hCB_1_ (black curves) to Site 5 (**A**,**D**,**G**,**J**) and Site 5.5 mutants (putative cholesterol binding sites; (**B**,**E**,**H**,**K**)) and Site 8 mutants (putative pregnenolone binding site; (**C**,**F**,**I**,**L**)) over a 25 min period. Data are pooled across five independent biological replicates and expressed as mean ± SEM. Data are expressed as ΔBRET ratio as matched vehicle conditions have been subtracted.

**Figure 5:**
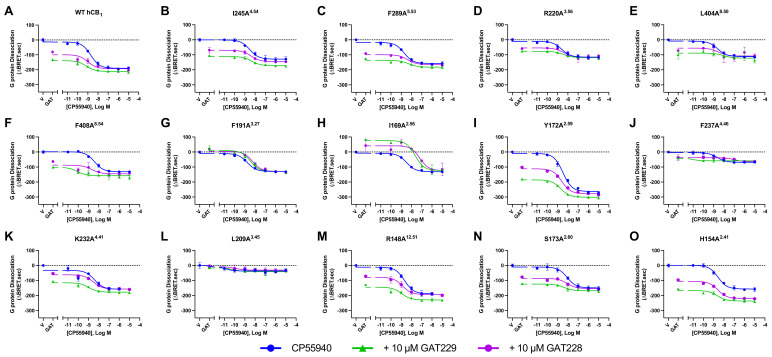
G_αi3_ protein dissociation HEK293 cells transiently transfected with WT hCB_1_ or mutant receptor to investigate allosteric modulation of CP55940. Concentration series of CP55940 alone (blue curves) or in the presence of 10 μM GAT229 (green curve) or 10 μM GAT228 (purple curve) showing G_αi3_ protein dissociation in HEK293 cells transiently expressing WT (**A**), I245A^4.54^ (**B**), F289A^5.53^ (**C**), R220A^3.56^ (**D**), L404A^8.50^ (**E**), F408A^8.54^ (**F**), F191A^3.27^ (**G**), I169A^2.56^ (**H**), Y172A^2.59^ (**I**), F237A^4.46^ (**J**), K232A^4.41^ (**K**), L209A^3.45^ (**L**), R148A^12.51^ (**M**), S173A^2.60^ (**N**), or H154A^2.41^ (**O**) over a 25 min period. Data are representative of five independent biological replicates and expressed as mean ± SD from technical triplicates within the same assay.

Y172A^2.59^ caused a significant increase in the efficacy (E_MAX_) of CP55940-induced G protein dissociation compared to WT hCB_1_ (Table 2, Figure 3, Figure 4 and Figure 5). In contrast, numerous mutant receptors resulted in a significant reduction in the efficacy (E_MAX_) of CP55940-induced G protein dissociation compared to WT hCB_1_ (namely R220A^3.56^, L404A^8.50^, F408A^8.54^, F191A^3.27^, I169A^2.56^, F237A^4.46^, L209A^3.45^, S173A^2.60^, and H154A^2.41^; Table 2, Figure 3, Figure 4 and Figure 5). CP55940 had reduced potency at S173A^2.60^ and I169A^2.56^, whereas CP55940 was equipotent at all other modified receptors (Table 2, Figure 5).

To assess the importance of specific residues within the six putative binding sites on allosteric agonism/inverse agonism, 10 μM of ZCZ011, GAT229, or GAT228 were tested at each mutant. Interestingly, all allosteric ligands appear to inhibit constitutive G protein dissociation (manifesting as inverse agonists) at F191A^3.27^, compared to inducing efficacious G protein dissociation at WT hCB_1_ (Table 3 and Figure 3). A similar trend was observed for I169A^2.56^, as GAT229 and GAT228 inhibited constitutive G protein dissociation (i.e., produced an inverse agonist-like response) compared to inducing efficacious G protein dissociation at WT hCB_1_ (Table 3 and Figure 3). A substantial reduction in G protein dissociation by ZCZ011 was also observed at I169A^2.56^; however, inverse agonism was not observed (Table 3, Figure 3). Maximal G protein dissociation by GAT229 and GAT228 was increased at Y172A^2.59^ compared to WT hCB_1_; however, ZCZ011 resulted in equivalent G protein dissociation (Table 3 and Figure 3). Similar to the results with CP55940, significant decreases in maximal G protein dissociation by all allosteric agonists were observed at F237A^4.46^ and L209A^3.45^ (Table 3 and Figure 3 and Figure 4). Both CP55940 and ZCZ011 had similar profiles across all mutants, with significant reductions in G protein dissociation being observed at R220A^3.56^, L404A^8.50^, F408A^8.54^, and H154A^2.41^ mutants, while these mutants did not alter G protein dissociation of GAT229 or GAT228 (Table 3 and Figure 3 and Figure 4).

### 2.3. Effect of hCB_1_ Mutants on Orthosteric Agonist-Induced G Protein Dissociation in Combination with Allosteric Ligands

To establish whether the modulatory effects of allosteric ligands were affected by mutagenesis of putative binding site residues, a high concentration (10 μM) of CP55940 alone was compared to 10 μM of CP55940 in the presence of 10 μM of ZCZ011. A complete concentration series of CP55940 alone, was also compared to a concentration series of CP55940 in the presence of 10 μM of GAT229 or GAT228, to evaluate changes in potency and efficacy of CP55940-induced G protein dissociation.

In WT hCB_1_ expressing cells, both ZCZ011 and GAT229 increased maximal CP55940-induced G protein dissociation (Table 2 and Table 3 and Figure 5). The ability of ZCZ011 (10 μM) to increase G protein dissociation by 10 μM of CP55940 was retained for all mutants except for I169A^2.56^, where in the presence of ZCZ011 (10 μM) CP55940-induced G protein dissociation was comparable for WT hCB_1_ and I169A^2.56^ (Table 2). GAT229 (10 μM) significantly increased efficacy of CP55940 at most modified receptors; however, this potentiation was lost at R220A^3.56^, L404A^8.50^, F191A^3.27^, I169A^2.56^, and F237A^4.46^ (Table 2 and Figure 5). In contrast, GAT228 (10 μM) did not alter CP55940-induced G protein dissociation in WT hCB_1_ expressing cells (Table 2, Figure 5). Interestingly, GAT228 (10 μM) significantly increased CP55940-induced G protein dissociation at F408A^8.54^, Y172A^2.59^, S173A^2.60^, or H154A^2.41^, indicating an increase in observed positive allosteric modulation (Table 2 and Figure 5). 

Neither GAT229 nor GAT228 increased the potency of CP55940-induced G protein dissociation at WT hCB_1_, in fact GAT228 caused a small but significant decrease in the potency of CP55940 (Table 2 and Figure 5). Interestingly, both GAT229 (10 μM) and GAT228 (10 μM) increased the potency of CP55940-induced G protein dissociation at Y172A^2.59^ and R148A^12.51^; however, these increases were less than 0.5 log units (Table 2 and Figure 5). Additionally, GAT229 (10 μM) significantly increased the potency of CP55940 at the S173A^2.60^ mutant (Table 2 and Figure 5). In contrast, the presence of 10 μM of GAT229 significantly decreased the potency of CP55940-induced G protein dissociation at both F191A^3.27^ and I169A^2.56^ mutant receptors (Table 2, Figure 5). Similarly, 10 μM of GAT228 significantly decreased the potency of CP55940-induced G protein dissociation at F289A^5.53^, L404A^8.50^, F191A^3.27^, and F237A^4.46^ mutants (Table 2 and Figure 5). As full concentration responses of CP55940 in combination with ZCZ011 were not performed, potency alterations were not considered.

### 2.4. Receptor Expression

To determine whether changes in G protein dissociation were in fact due to altered ligand binding rather than changes in receptor expression, immunocytochemistry was performed to quantify both surface and total receptor expression for each mutant (Table 4).

Receptor expression (both surface and total) was found to be equivalent at most modified receptors relative to WT hCB_1_ (Table 4). However, significantly decreased surface receptor expression was observed for L209A^3.45^, F237A^4.46^ and R148A^12.51^ mutants, with L209A^3.45^ also having decreased total receptor expression compared to WT hCB_1_ (Table 4). Although statistical significance was not reached, the Y172A^2.59^ mutant had higher surface and total receptor expression relative to WT hCB_1_ (Table 4). 

## 3. Discussion

PAMs at CB_1_ have been found to be promising in the treatment of neurodegenerative and pain-related disorders as they produce therapeutic outcomes in the absence of the on-target adverse effects classically associated with activation of CB_1_ [8,11,12,13,14,15,16,17,18]. Although PAMs demonstrate promising therapeutic utility in vivo, structural modifications to enhance allosteric modulation and further investigation into the binding site will provide key information for future drug development.

To assess residues critical for allosteric agonism and/or allosteric modulation we mutated 14 key residues within six putative binding sites. These included Site 2—the putative GAT229 binding site [22], Site 4—the putative GAT228 binding site [22], Site 3—the crystal structure ZCZ011 binding site [23], Site 8—the putative pregnenolone binding site [24], and Sites 5/5.5—two putative cholesterol binding sites. While the model was able to identify previously reported putative allosteric binding sites, a key feature was the elucidation of novel putative allosteric binding sites—Sites 5/5.5, which have previously only been defined as cholesterol binding sites [26]. As SiteMap identifies these as putative allosteric binding sites, this indicates an increased likelihood that cholesterol and/or other ligands may interact here. Allosteric modulators have been proposed to bind to multiple other cholesterol binding sites on hCB_1_ [22,27]. Membrane cholesterol has been suggested to be a key mediator of GPCR signalling, specifically regarding the development of tolerance to therapeutic effects [28]. This indicates that the role of cholesterol in allosteric modulation of hCB_1_ should be further investigated, specifically whether hCB_1_ allosteric ligands compete with endogenous cholesterol for cholesterol binding sites.

Efficacy of both CP55940- and allosteric agonist-induced G protein dissociation was significantly reduced at two mutants (F237A^4.46^ and L209A^3.45^; Table 2 and Table 3, and Figure 3 and Figure 4). Analysis of cell surface receptor expression suggests that the consistent decrease in G protein dissociation by both the L209A^3.45^ and F237A^4.46^ mutants is due to a significant reduction in surface expression (Table 4), although it is also likely that F237^4.46^ may have a role in classical activation of hCB_1_ [23]. F237^4.46^ has recently been proposed to play a key role in activation of hCB_1_, as F155^2.42^ in the inactive state forms a stabilising network of interactions with the interior of the transmembrane bundle. F237^4.46^ could potentially attract F155^2.42^ via π-bonding to adopt its outward-facing rotamer, loosening the bundle and thus facilitating the outward movement of TM6 and receptor activation [23]. Furthermore, mutation of F237^4.46^ to a lysine (F237L) was found to stabilise an inactive conformation and inhibit agonist-induced activation while not affecting the affinity of CP55940 [29]. Upon receptor activation, F155^2.42^ moves from facing the G protein cavity to face the extrahelical cavity and interacts with L209^3.45^ [29]. It is possible that this interaction is disrupted in L209A^3.45^, therefore restricting receptor activation by both orthosteric and allosteric ligands (Figure 3 and Figure 4, and Table 2 and Table 3). In combination with decreased cell surface expression this is likely to explain the decrease in G protein dissociation observed at L209A^3.45^ and F237A^4.46^ (Figure 3 and Figure 4, and Table 2 and Table 3). CP55940 was observed to have decreased potency at S173A^2.60^ compared to WT hCB_1_, potentially due to loss of a hydrogen bond between the side chain of S173^2.60^ and the hydroxypropyl group of CP55940 in WT hCB_1_ [23,27]. As these three mutants have altered CP55940 signalling, changes in G protein dissociation by allosteric agonists are more difficult to interpret, as these residues are likely to have a structural role in the activation of the receptor regardless of the mechanism of activation. Therefore, the reductions in efficacy observed for allosteric ligands at F237A^4.46^ and L209A^3.45^ can be attributed to conformational restrictions of hCB_1,_ and decreased cell surface expression as opposed to F237^4.46^ and L209^3.45^ being key residues within the allosteric binding site (Figure 3 and Figure 4, and Table 2 and Table 3). Interestingly, an increase in G protein dissociation by CP55940, GAT229, and GAT228 was observed at Y172A^2.59^, while this mutant had no effect on G protein dissociation by ZCZ011 (Table 2 and Table 3, and Figure 3, Figure 4 and Figure 5). Our model suggests Y172 undergoes a rotamer shift during receptor activation (unpublished data). Its distance from F191 increases (from ~9 to 13 Å) and it may form H-bonds with D176, facilitating the clockwise rotation and inward kinking of the extracellular region of TM2 needed for receptor activation. Mutant Y172A^2.59^ reduces the energy barrier for this process, therefore potentially facilitating receptor activation and increasing G protein dissociation.

**Figure 6:**
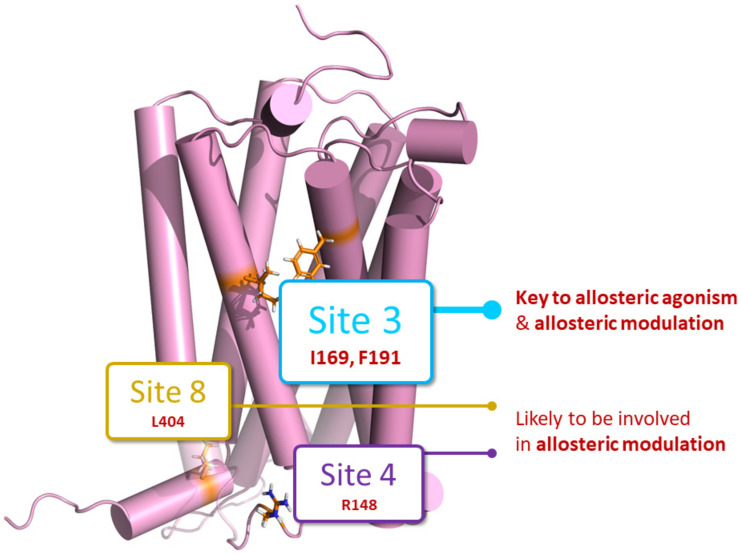
Summary figure showing key binding sites for allosteric agonism and/or allosteric modulation. Site 3 (proposed ZCZ011 binding site, [23]) was found to be crucial for allosteric agonism, as G protein dissociation was abolished at two mutants within this site (I169A^2.56^ and F191A^3.27^). Site 3, along with Site 8 (proposed pregnenolone binding site [24]) and Site 4 (proposed GAT228 binding site [22]), were identified as sites likely to be involved in allosteric modulation, as the positive allosteric modulatory ability of ZCZ011 and GAT229 was lost at mutants within these sites.

Mutagenesis studies in this manuscript indicate it is likely that all allosteric ligands tested here (GAT229, GAT228, and ZCZ011) bind to a significant extent at the previously reported allosteric binding site identified when hCB_1_ was crystallised bound to CP55940 and (*S*)-ZCZ011 [23]. This is due to complete abolishment of G protein dissociation by allosteric agonists when residues within this proposed binding site (F191^3.27^ and I169^2.56^) were mutated (Figure 3, Figure 4 and Figure 6, and Table 3). The thiophene ring of ZCZ011 has been proposed to hydrogen bond and form π-π stacking interactions with F191^3.27^ upon binding, stabilising the upward/active conformation of TMH3 [23]. ZCZ011 was also found to interact directly with I169^2.56^, therefore it is likely that allosteric ligands require interactions with F191^3.27^ and I169^2.56^ to induce allosteric G protein dissociation [23]. Interestingly, the crystal structure showed that (*S*)-ZCZ011 directly interacts with I245^4.54^, however, allosteric agonist-induced G protein dissociation was not altered at I245A^4.54^ by any allosteric agonist [23] (Figure 3 and Figure 4, and Table 3). Compared to F191^3.27^ and I169^2.56^, we found that I245^4.54^ has a lower per residue interaction energy, indicating that it may be less involved in the binding of allosteric ligands. It is also possible that each allosteric agonist investigated may have unique interactions with the residues within this binding site, as some subtle differences were observed. Both GAT229 and GAT228 caused significant inhibition of constitutive G protein dissociation (resulting in inverse agonism) at the I169A^2.56^ mutant, whereas no inverse agonism was observed in response to ZCZ011 (Figure 3 and Table 3). It is not a surprise that the allosteric ligands tested here are likely to be binding to the same binding site given the structural similarities and comparable in vitro pharmacological profiles observed (Green et al., 2023). While mutagenesis of residues within this site abolished the efficacy of allosteric agonist-induced G protein dissociation, it is important to note that we cannot make observations regarding the affinity of allosteric agonists for altered receptors, as to do so would require radioligand binding experiments. Furthermore, residues identified as crucial for allosteric agonist-induced G protein dissociation may differ from residues crucial for activation of other downstream signalling pathways, such as ERK1/2 phosphorylation, β arrestin recruitment, and internalisation of hCB_1_; therefore, further in vitro characterisation is required.

Positive allosteric modulation of orthosteric agonist efficacy, observed as the potentiation of maximal CP55940-induced G protein dissociation, was observed with ZCZ011 and GAT229 (but not GAT228) in combination with CP55940 at WT hCB_1_ (Figure 5 and Table 2 and Table 3). As GAT228 did not significantly increase the efficacy or potency of CP55940-induced G protein dissociation, it behaves exclusively as an allosteric agonist with no modulatory activity in this system. The ability of ZCZ011 to potentiate maximal CP55940-induced G protein dissociation was conserved in all mutants, excluding I169A^2.56^ (Table 3). In contrast, the modification of multiple residues (R220A^3.56^, L404A^8.50^, F191A^3.27^, I169A^2.56^, and F237A^4.46^) abolished the ability of GAT229 to increase the efficacy of CP55940-induced G protein dissociation (Table 2 and Figure 5). However, as F237A^4.46^ was found to have decreased cell surface expression and is likely to have a role in the activation of hCB_1_, it is unlikely that this reduction indicates that GAT229 interacts directly with F237^4.46^. These results may suggest that some allosteric modulators (*e.g.,* GAT229) may bind to more than one site to exert their allosteric modulatory effects, including Sites 3, 4, and 8 in this study (Figure 6). However, I169^2.56^ is indicated to play a crucial role in allosteric modulation of CP55940 for both ZCZ011 and GAT229, as neither allosteric ligand acted as a PAM at I169A^2.56^.

## 4. Materials and Methods

### 4.1. Drugs

CP55940 (stored at 10 mM in EtOH), GAT229, and GAT228 (stored at 31.6 mM in DMSO) were purchased from Cayman Chemical Company (Ann Arbour, MI, USA). Racemic ZCZ011 was provided as a generous gift from Professor Ruth Ross (University of Toronto) and stored at 10 mM in DMSO. All drugs were aliquoted into single use aliquots and stored at −80 °C.

### 4.2. Cell Lines and Maintenance

Mutagenesis studies using the pIRES G protein dissociation assay [30] were performed using human embryonic kidney 293 (HEK293) wild-type cells transiently transfected with pplss-3HA-hCB_1_ [31] or modified receptor. All cells were cultured in Dulbecco’s Modified Eagle’s Medium (DMEM) supplemented with 10% fetal bovine serum (FBS), grown in 75 m^2^ vented-cap flasks, and maintained in a 37° C incubator at 5% CO_2_.

### 4.3. Identification of Putative PAM Binding Sites

Using our in-house hCB_1_ model based on previously available crystal and cryo-EM structures of hCB_1_ and refined with published NMR studies, Schrödinger Maestro was used to optimise the H-bond networks of polar side chains. Restrained minimisation was then used to settle any resulting clashes, and the final protein was submitted to the Sitemap program (Schrödinger Release 2023-3: SiteMap, Schrödinger, LLC, New York, NY, USA, 2023) [32,33]. Sitemap identified ten putative binding sites in or around the surface of the receptor. These included the previously suggested GAT228, GAT229, and ZCZ011 binding sites in addition to sites at which cholesterol is often co-crystallised in XRD structures of hCB_1_, and the negative allosteric modulator (NAM) binding site at which pregnenolone is reported to bind [24]. It should be noted that for this study of PAM binding sites, a receptor model in the active state was used. Residues involved in the proposed pregnenolone binding site may therefore differ in conformation to when the receptor is in the inactive state. Additional novel binding sites were also identified. One Sitemap result corresponding to the transducer-binding cavity [34] was excluded from further analysis, as small molecule binding at this site would inhibit the binding of G proteins and/or β-arrestins and is therefore not a likely candidate for PAM binding.

Next, previously reported allosteric ligands GAT228, GAT229, (*S*)-ZCZ011, and (*R*)-ZCZ011 were docked to each site, using Schrodinger Glide Induced Fit Docking. The docking scores from these runs were plotted and compared to assist in ranking the binding sites, and from each site a representative binding site conformation was selected for further docking. Each literature compound was then docked using regular Glide docking to each binding site, and the per-residue interaction energies were tabulated to assist in the identification of key residues for subsequent mutagenesis studies. Additionally, residue conservation data was retrieved from GPCR-DB to further inform residue selection.

### 4.4. QuikChange^®^ Mutagenesis—Development of hCB_1_ Mutants

Mutants of pplss-3HA-hCB_1_ (WT, pEF-V4-HisA (pEF4a) construct) were generated using a modified QuikChange^®^ (Stratagene, San Diego, CA, USA) site-directed mutagenesis approach using KAPA HiFi Hotstart Polymerase (KAPA Biosystems, Roche, Basel, Switzerland). Briefly, single-stranded primers used to modify one or two base pairs within the target amino acid using PCR were purchased from Integrated DNA Technologies (IDT, Coralville, IA, USA). All pplss-hCB_1_ mutants were generated using the pplss-3HA-hCB_1_ pEF4a as parental DNA. PCR products were generated using recommended cycling conditions and treated with *Dpn1* (New England Biolabs, Ipswich, MA, USA) overnight at 37° C to digest methylated parental DNA. PCR products were electrophoresed and run on an agarose gel (1% (*w*.*v*^−1^) agarose, HydraGene, Piscataway, NJ, USA) containing 0.25 μg/mL ethidium bromide (Sigma-Aldrich, St. Louis, MO, USA). Gels were run in 40 mM TRIS-acetate buffer containing 20 mM EDTA (pH 8.3) at 100 V for 30 min, maximum current and included a 1kb+ DNA ladder (Invitrogen, Thermo Fisher Scientific, Carlsbad, CA, USA) for reference. Gels were imaged using an UVTech Alliance Q9 Mini transilluminator (UVTech Alliance, Cambridge, UK). Digested products were then used to transform NEB 5α competent *E. coli* cells in accordance with manufacturer’s instructions. Briefly, transformed PCR products were grown overnight on 8.5 mL Luria Broth agar plates (containing 100 μg/mL ampicillin) at 37° C. Single bacterial colonies were picked and inoculated in Luria Broth (containing 100 μg/mL ampicillin) and grown overnight in a shaking incubator at 37° C. Plasmids were then harvested and purified using a Qiagen miniprep kit (Qiagen, Hilden, Germany) and validated using sequencing (Genetics Analysis Service, University of Otago, Dunedin, New Zealand).

### 4.5. pIRES G Protein Dissociation Assay

Dissociation of the G protein heterotrimer, specifically G_αi1_, G_β1_, and G_γ2_, was investigated using a bioluminescence resonance energy transfer (BRET) assay with the pIRES biosensor first described by [30] and adapted by [35]. This assay was used for mutagenesis studies probing the binding site of allosteric modulators alone and in combination with CP55940.

Briefly, 3 × 10^6^ WT HEK293 cells were seeded into 10 cm culture dishes (Corning, Corning, NY, USA; one dish for the control receptor and one for each modified receptor) and incubated at 37 °C for 24 h to gain 50–60% confluency. Culture medium was then replaced and transfection mixtures containing 3 μg pIRES vector and 1 μg receptor (either pplss-hCB_1_ or modified receptor) were prepared (4 μg total DNA). Plasmids were initially diluted in sterile Milli-Q water before being diluted in OptiMEM reduced serum medium (Thermo Fisher Scientific, Waltham, MA, USA) and combined with 45 μg polyethylenimine (PEI)-max (1:9 DNA:PEI-max ratio) and incubated at room temperature for 20 min. Transfection mixtures were then added to cells via dropwise addition, and cells were incubated at 37 °C for 24 h. Transfected cells were lifted and plated at 50,000 cells/well into poly-d-lysine (PDL; Sigma-Aldrich) coated white 96-well CulturPlates (PerkinElmer, Waltham, MA, USA) and incubated for a further 24 h at 37 °C.

To assay, the culture medium was aspirated and cells were washed with phosphate buffered saline (PBS) and replaced with BRET assay medium (phenol red free DMEM containing 25 mM HEPES and supplemented with 1 mg/mL bovine serum albumin; BSA, MP Biomedicals, Auckland, New Zealand) for 30 min prior to drug addition (serum starve). Drugs were prepared at 10 × concentration in BRET assay medium, combined in a polypropylene V-bottom plate, and incubated at 37° C prior to drug stimulation. Coelenterazine-h (Nanolight Technology, Prolume Ltd, Pinetop, AZ, USA) was prepared at 10 × concentration in BRET assay medium (final concentration 5 μM) and dispensed to cells immediately prior to drug stimulation. Plates were transferred to the LUMIstar Omega plate reader (BMG Labtech GmbH, Ortenburg, BW, Germany) and luminescence (475 and 535 nm) was detected simultaneously in the dark with BRET1 filters for approximately 5 min to establish a baseline BRET ratio. Drugs were added to cells, plates were returned to the LUMIstar and luminescence was detected for a further 25 min. BRET ratios (475/535) were calculated in Omega MARS software v 3.32, exported, and data were analysed using GraphPad Prism v8. Baseline correction was performed using an in-built function in GraphPad Prism, subtracting vehicle BRET ratios from matched conditions. Area under the curve (AUC) analysis was performed in GraphPad Prism using an in-built function to obtain concentration response data.

### 4.6. Immunocytochemistry for Receptor Expression

Immunocytochemistry was used to quantify both surface and total receptor expression of pplss-hCB_1_-WT and mutant receptors [36]. Transfected HEK293 WT cells from pIRES G protein dissociation were plated into PDL-coated clear Costar 96-well plates at a density of 50,000 cells/well and incubated for 24 h at 37 °C. For detection of surface level receptor expression (conducted on live cells), plating medium was aspirated and washed with BRET assay medium before the addition of 35 µL primary mouse anti-HA.11 clone 16B12 monoclonal antibody (BioLegend, San Diego, CA, USA; cat. No. 901503; RRID: AB_2565005) diluted in BRET assay medium (1:500). Cells were then placed on a plate rocker and incubated at room temperature for 30 min. Primary antibody was aspirated, cells were washed with BRET assay medium, and fixed for 10 min in 4% (w/v) PFA (Sigma-Aldrich, St. Louis, MO, USA) in 0.1 M phosphate buffer. For detection of total receptor expression (conducted on cells postfixation in PFA), plating medium was aspirated, and cells were fixed for 10 min in 4% PFA. Following fixation, all cells (both those for surface and total receptor expression) were washed twice with PBS, and cells for surface receptor were washed with PBS containing 0.2% Triton X-100 (PBS-T). Primary mouse anti-HA.11 antibody was diluted 1:1000 in PBS supplemented with 1% goat serum, 0.2% Triton X-100, and 0.4 mg/mL Merthiolate (immunobuffer), added to cells (for detection of total receptor expression) and incubated on a plate rocker at room temperature for 3 h. Primary antibody was then aspirated, and cells were washed with PBS-T.

Secondary antibody, Alexa Fluor goat anti-mouse highly cross-adsorbed 594 (Invitrogen, Thermo Fisher Scientific, Carlsbad, CA, USA; cat. No. A11032; RRID: AB_2534091) was diluted in immunobuffer (1:400), added to all cells and incubated for 3 h at room temperature on a plate rocker. Secondary antibody was aspirated, and cells were washed with PBS-T. Cell nuclei were stained using Hoechst 33258 (4 mg/mL in MilliQ water; Sigma-Aldrich, St. Louis, MO, USA) diluted in PBS-T (1:500) and incubated on a plate rocker for 20 min at room temperature. Cells were then washed twice with PBS-T and stored in PBS-T supplemented with 0.4% Merthiolate. Cells were then imaged using a Phenix Opera High Content System at the Otago Micro and Nanoscale Imaging (OMNI) facility, using a 20× objective lens capturing 24 sites/well. Dichroic filters were used to measure both Hoechst 33258 (excitation 375 nm, emission 430–480 nm) and Alexa Fluor 594 (excitation 561 nm, emission 570–630 nm) were used to capture images. Quantitative immunocytochemistry analysis was performed using Signals Image Artist (version 1.0), whereby the number of nuclei were counted and intensity of Alexa Fluor 594 staining (within set thresholds to define real staining from background) was recorded. Total fluorescence per well was divided by the number of nuclei per well to determine ‘integrated intensity per well’. Statistical analysis was performed using GraphPad Prism v9.

### 4.7. Data and Statistical Analysis

All data analysis was performed using GraphPad Prism v8. Sigmoidal concentration series were generated by performing AUC analysis and fit using three-parameter nonlinear regression curves. Statistical analyses were performed using data interpolated from nonlinear regression curves using parameters derived from five independent biological replicates. Both one- and two-way repeated measures ANOVA and paired t-tests were used where appropriate (specified in-text), and posthoc tests, specified in text, were performed when significance was reached (*p* < 0.05). Time course data presented in this manuscript are pooled data (*n* = 5) and expressed as mean ± SEM. Concentration response data are representative, data expressed as mean ± SD from technical triplicates, to avoid misestimation of parameters from combined/pooled data [37].

## 5. Conclusions

The most significant finding of this study was that the allosteric ligands investigated (ZCZ011, GAT229, and GAT228) are likely to exert some or all of their agonist effects via the ZCZ011 binding site established by crystallography [23]. Mutation of two key amino acid residues (I169A^2.56^ and F191A^3.27^) within this site (Site 3) led to abolishment of allosteric agonist-induced G protein dissociation. This is in contrast with previously suggested binding sites for allosteric ligands, where selective binding at Site 4 (GAT228) or Site 2 (GAT229) has been proposed as a mechanism to elicit the distinct pharmacological responses of GAT228 and GAT229 [22]. However, following investigation using assays with greater sensitivity, GAT229 has been found to be an efficacious allosteric agonist (indistinguishable from GAT228), aligning with our finding that both these ligands are likely to bind to Site 3 to induce allosteric activation. In combination with the orthosteric agonist CP55940, reduction in the allosteric modulatory activity of GAT229 following mutation of other sites (Site 4, 5.5, and 8) suggests that observed PAM activity for a single ligand, may in fact be due to a net effect of binding at multiple sites on the receptor. 

These results suggest that future drug development directed towards pure PAM activity should avoid allosteric binding at the ZCZ011 crystal structure binding site, as interaction with this site is likely to result in allosteric agonism [23]. Elucidation of these subtleties in the hCB_1_ PAM binding sites should facilitate development of PAMs to elicit specific receptor responses, based on targeted interactions with specific residues, and increased understanding of their individual signalling contributions.

## Figures and Tables

**Table 1:** Allosteric binding site residues selected for mutagenesis.

Binding Site	Proposed Ligand	Mutation	BW Number ^a^	% Conserved ^b^	Consensus Residue ^b^	% CB_1_ ^c^
Site 2	GAT229	Y172A	2.59 × 59	36	F	1
		S173A	2.60 × 60	12	V	1
Site 2/3		F191A	3.27 × 27	29	L	13
Site 3	ZCZ011	F191A	3.27 × 27	29	L	13
		I169A	2.56 × 56	24	T	6
		I245A	4.54 × 54	28	L	10
Site 4	GAT228	R148A	12.51 × 51	28	Gap ^d^	-
		H154A	2.41 × 41	22	I	1
		F237A	4.46 × 46	29	I	1
Site 5	Cholesterol	K232A	4.41 × 41	25	R	8
		L209A	3.45 × 45	38	A	13
Site 5.5	Cholesterol	F289A	5.53 × 53	30	I	5
		R220A	3.56 × 56	32	H	13
Site 8	Pregnenolone	L404A	8.50 × 50	60	F	9
		F408A	8.54 × 54	33	F	35

^a^ Ballesteros–Weinstein numbering [25]. ^b^ The percentage of Family A GPCRs that share the consensus residue in this position. ^c^ The percentage of Family A GPCRs that share the same residue as CB_1_ in this position. ^d^ CB_1_ has an additional residue in this position.

**Table 2:** G protein dissociation by CP55940 in the absence and presence of GAT229 or GAT228 at putative PAM binding site mutants ^a^.

		CP55940 Alone		CP55940 + 10 μM GAT229		CP55940 + 10 μM GAT228	
Site	Mutation	pEC_50_	E_MAX_	pEC_50_	E_MAX_	pEC_50_	E_MAX_
-	WT ^b^	8.59 ± 0.06	−184.1 ± 3.8	8.65 ± 0.13	−205.1 ± 7.8 ^†^	8.42 ± 0.08 ^†^	−194.2 ± 7.3
Site 2	Y172A^2.59^	8.46 ± 0.02	−273.2 ± 6.9 *	8.62 ± 0.03 ^†^	−309.0 ± 3.5 ^†^	8.60 ± 0.04 ^†^	−298.9 ± 5.4 ^†^
	S173A^2.60^	8.00 ± 0.08 *	−143.0 ± 5.7 *	8.20 ± 0.08 ^†^	−186.4 ± 7.5 ^†^	7.91 ± 0.10	−176.1 ± 6.9 ^†^
Site 2/3	F191A^3.27^	8.87 ± 0.06	−117.3 ± 6.1 *	8.46 ± 0.07 ^†^	−119.1 ± 7.8	8.33 ± 0.06 ^†^	−123.9 ± 7.4
Site 3	I169A^2.56^	8.20 ± 0.08 *	−110.7 ± 10.3 *	7.65 ± 0.03 ^†^	−109.7 ± 8.3	7.76 ± 0.16	−117.0 ± 7.4
	I245A^4.54^	8.51 ± 0.07	−150.1 ± 9.8	8.58 ± 0.18	−182.9 ± 7.0 ^†^	8.26 ± 0.16	−163.7 ± 8.2
Site 4	R148A^12.51^	8.46 ± 0.10	−175.6 ± 11.4	8.93 ± 0.06 ^†^	−243.9 ± 5.4 ^†^	8.67 ± 0.09 ^†^	−218.2 ± 8.5
	H154A^2.41^	8.60 ± 0.10	−151.8 ± 5.9 *	9.06 ± 0.40	−210.6 ± 10.1 ^†^	8.53 ± 0.13	−200.5 ± 11.6 ^†^
	F237A^4.46^	8.60 ± 0.11	−69.8 ± 6.7 *	8.60 ± 0.70	−67.4 ± 8.3	7.52 ± 0.30 ^†^	−69.4 ± 6.4
Site 5	K232A^4.41^	8.61 ± 0.05	−160.6 ± 7.6	8.58 ± 0.20	−194.9 ± 9.0 ^†^	8.45 ± 0.11	−181.9 ± 8.6
	L209A^3.45^	ND	−39.8 ± 1.1 *	ND	−51.9 ± 2.8 ^†^	ND	−43.2 ± 4.5
Site 5.5	F289A^5.53^	8.62 ± 0.11	−145.4 ± 5.7	7.97 ± 0.22	−161.1 ± 6.5 ^†^	8.17 ± 0.10 ^†^	−156.7 ± 5.3
	R220A^3.56^	8.58 ± 0.08	−110.8 ± 2.8 *	8.76 ± 0.34	−144.6 ± 10.4	8.41 ± 0.14	−140.1 ± 13.5
Site 8	L404A^8.50^	8.66 ± 0.05	−103.1 ± 3.6 *	8.36 ± 0.45	−122.3 ± 7.0	8.39 ± 0.12 ^†^	−115.1 ± 8.3
	F408A^8.54^	8.56 ± 0.07	−128.6 ± 12.1 *	8.98 ± 0.38	−154.0 ± 14.5 ^†^	8.11 ± 0.20	−148.4 ± 11.4 ^†^

^a^ Data are presented as mean ± SEM of five independent biological replicates, with E_MAX_ defined as the top of the curve (maximal response, ∆BRET.sec). Statistical tests to compare the response of CP55940 at each different mutant compared to WT hCB_1_ were performed in GraphPad Prism using a repeated measures one-way ANOVA with Dunnett’s multiple comparisons test (* < 0.05). Statistical tests to compare CP55940 (alone) to CP55940 in the presence of allosteric ligand at matched receptor mutants were performed in GraphPad Prism using a paired t-test (^†^ < 0.05). ND indicates values that were not determined due to inactivity/poorly defined potency. ^b^ Data are presented as mean ± SEM of ten independent biological replicates; however, statistical analysis to compare CP55940 at WT hCB_1_ to mutated receptors was performed using matched data from five independent biological replicates.

**Table 3:** G protein dissociation by cannabinoid ligands alone and in combination at putative allosteric binding site mutants ^a^.

Site	Mutation	10 μM CP55940	10 μM GAT229	10 μM GAT228	10 μM ZCZ011	10 μM CP55940 + 10 μM ZCZ011
-	WT ^b^	−188.7 ± 3.6	−148.6 ± 7.2	−105.7 ± 7.2	−167.3 ± 3.2	−215.9 ± 2.9 ^†^
Site 2	Y172A^2.59^	−284.7 ± 8.4 *	−207.0 ± 7.1 *	−133.4 ± 9.8 *	−142.1 ± 14.4	−305.2 ± 5.7 ^†^
	S173A^2.60^	−146.2 ± 5.5 *	−137.3 ± 4.8	−105.5 ± 7.6	−144.2 ± 6.8	−186.8 ± 5.5 ^†^
Site 2/3	F191A^3.27^	−117.8 ± 7.5 *	22.1 ± 6.4 *	19.5 ± 11.9 *	35.0 ± 8.4 *	−134.7 ± 12.4 ^†^
Site 3	I169A^2.56^	−113.1 ± 11.7 *	54.8 ± 7.5 *	30.0 ± 5.9 *	−20.0 ± 9.0 *	−120.0 ± 10.9
	I245A^4.54^	−154.9 ± 10.6	−121.6 ± 8.0	−90.3 ± 8.5	−130.4 ± 12.9	−183.2 ± 7.2 ^†^
Site 4	R148A^12.51^	−179.5 ± 12.2	−148.5 ± 7.7	−102.7 ± 11.8	−150.1 ± 10.2	−237.3 ± 11.6 ^†^
	H154A^2.41^	−155.3 ± 6.5 *	−154.6 ± 7.8	−101.6 ± 6.9	−129.4 ± 5.1 *	−189.5 ± 8.5 ^†^
	F237A^4.46^	−69.8 ± 6.5 *	−52.4 ± 5.2 *	−40.8 ± 2.8 *	−64.2 ± 4.1 *	−85.2 ± 9.9 ^†^
Site 5	K232A^4.41^	−167.7 ± 9.6	−134.5 ± 7.9	−94.0 ± 10.5	−140.0 ± 7.3	−194.9 ± 7.2 ^†^
	L209A^3.45^	−42.7 ± 2.8 *	−25.1 ± 3.5 *	−19.0 ± 3.3 *	−34.4 ± 2.9 *	−71.3 ± 4.2 ^†^
Site 5.5	F289A^5.53^	−148.1 ± 5.2	−123.6 ± 5.9	−97.4 ± 7.2	−135.5 ± 6.3	−167.8 ± 6.9 ^†^
	R220A^3.56^	−112.1 ± 2.9 *	−102.9 ± 9.9	−85.2 ± 13.6	−95.7 ± 7.7 *	−137.8 ± 5.0 ^†^
Site 8	L404A^8.50^	−110.3 ± 3.7 *	−88.9 ± 6.2	−63.2 ± 5.6	−85.2 ± 7.3 *	−133.5 ± 5.6 ^†^
	F408A^8.54^	−131.7 ± 10.4 *	−110.0 ± 8.6	−90.3 ± 8.3	−116.9 ± 10.1 *	−159.7 ± 13.4 ^†^

^a^ Data are presented as mean ± SEM of five independent biological replicates, with data as AUC of 10 μM compound (maximal response). Statistical tests to compare the response of each ligand at each different mutant compared to WT hCB_1_ were performed in GraphPad Prism using a repeated measures one-way ANOVA with Dunnett’s multiple comparisons test (* < 0.05). Statistical tests to compare 10 μM CP55940 (alone) to CP55940 in the presence of 10 μM ZCZ011 at matched receptor mutants were performed in GraphPad Prism using a paired t-test (^†^ < 0.05). ^b^ As WT hCB_1_ was included as the control on each experimental day data are presented as mean ± SEM of ten independent biological replicates; however, statistical analysis to compare compounds at WT hCB_1_ to mutant receptors were performed using matched data from each experimental day. Therefore, five independent biological replicates for WT hCB_1_ and mutant receptors were utilised for statistical analysis.

**Table 4:** Receptor expression for WT hCB_1_ transfected cells compared to mutant hCB_1_ receptors ^a^.

Site	Mutant	Surface Receptor Expression	Total Receptor Expression
-	WT ^b^	224.5 ± 37.7	895.1 ± 141.3
Site 2	Y172A^2.59^	482.7 ± 104.8	1568 ± 448.5
	S173A^2.60^	313.2 ± 62.7	1477 ± 425.9
Site 2/3	F191A^3.27^	82.9 ± 43.6	620.2 ± 214.6
Site 3	I169A^2.56^	88.9 ± 33.8	525.5 ± 231.3
	I245A^4.54^	117.9 ± 60.1	631.2 ± 226.6
Site 4	R148A^12.51^	125.7 ± 34.4 *	966.5 ± 351.3
	H154A^2.41^	217.8 ± 70.1	1047 ± 394.8
	F237A^4.46^	71.5 ± 22.9 *	1117 ± 354.8
Site 5	K232A^4.41^	207.4 ± 50.5	1154 ± 351.2
	L209A^3.45^	18.7 ± 17.4 *	532.2 ± 173.6 *
Site 5.5	F289A^5.53^	217.4 ± 93.5	1043 ± 340.5
	R220A^3.56^	160.2 ± 56.3	813.6 ± 192.8
Site 8	L404A^8.50^	164.1 ± 126.2	389.7.1 ± 154.2 *
	F408A^8.54^	110.9 ± 55.6	634.9 ± 257.9

^a^ Data are presented as mean ± SEM of five independent biological replicates, with data as integrated intensity per cell (AU) of each receptor. Statistical tests to compare the receptor expression of each different mutant compared to WT hCB_1_ was performed in GraphPad Prism using a repeated measures one-way ANOVA with Dunnett’s multiple comparisons test (* < 0.05). ^b^ As WT hCB_1_ was included as the control on each experimental day, data are presented as mean ± SEM of ten independent biological replicates; however, statistical analysis to compare receptor expression of WT hCB_1_ to mutant receptors were performed using matched data from each experimental day. Therefore, five independent biological replicates for WT hCB_1_ and mutant receptors were utilised for statistical analysis.

## Data Availability

Data supporting the findings of this study are available from the corresponding author upon reasonable request.

## Supplementary Materials

The following supporting information can be downloaded at https://www.mdpi.com/article/10.3390/ph17020154/s1, An Excel file detailing the per-residue interaction energies for the top residues within each identified putative binding site is supplied.
